# Skin Immunization Obviates Alcohol-Related Immune Dysfunction

**DOI:** 10.3390/biom5043009

**Published:** 2015-11-06

**Authors:** Rhonda M. Brand, John Mark Stottlemyer, Rachel A. Cline, Cara Donahue, Jaideep Behari, Louis D. Falo

**Affiliations:** 1 Department of Dermatology, School of Medicine, University of Pittsburgh, Pittsburgh, PA 15213, USA; E-Mails: jms176176@gmail.com (J.M.S.); rachel.a.cline@gmail.com (R.A.C.); cmdst36@pitt.edu (C.D.); lof2@pitt.edu (L.D.F.); 2 Division of Gastroenterology, Hepatology and Nutrition, Department of Medicine, School of Medicine, University of Pittsburgh, Pittsburgh, PA 15213, USA; E-Mail: jab31@pitt.edu; 3 Magee Women’s Research Institute, Pittsburgh, PA 15213, USA; 4 Department of Bioengineering, University of Pittsburgh, Pittsburgh, PA 15213, USA; 5 Clinical and Translational Science Institute, School of Medicine, University of Pittsburgh, Pittsburgh, PA 15213, USA; 6 The University of Pittsburgh Cancer Institute, Pittsburgh, PA 15232, USA; 7 The McGowan Institute for Regenerative Medicine, School of Medicine, University of Pittsburgh, Pittsburgh, PA 15219, USA

**Keywords:** skin immunity, vaccination, alcohol, ethanol, intradermal immunization, skin, Lieber-DeCarli, Meadows-Cook

## Abstract

Alcoholics suffer from immune dysfunction that can impede vaccine efficacy. If ethanol (EtOH)-induced immune impairment is in part a result of direct exposure of immune cells to EtOH, then reduced levels of exposure could result in less immune dysfunction. As alcohol ingestion results in lower alcohol levels in skin than blood, we hypothesized that the skin immune network may be relatively preserved, enabling skin-targeted immunizations to obviate the immune inhibitory effects of alcohol consumption on conventional vaccines. We employed the two most common chronic EtOH mouse feeding models, the liver-damaging Lieber-DeCarli (LD) and liver-sparing Meadows-Cook (MC) diets, to examine the roles of EtOH and/or EtOH-induced liver dysfunction on alcohol related immunosuppression. Pair-fed mice were immunized against the model antigen ovalbumin (OVA) by DNA immunization or against flu by administering the protein-based influenza vaccine either systemically (IV, IM), directly to liver (hydrodynamic), or cutaneously (biolistic, ID). We measured resulting tissue EtOH levels, liver stress, regulatory T cell (Treg), and myeloid-derived suppressor cell (MDSC) populations. We compared immune responsiveness by measuring delayed-type hypersensitivity (DTH), antigen-specific cytotoxic T lymphocyte (CTL), and antibody induction as a function of delivery route and feeding model. We found that, as expected, and independent of the feeding model, EtOH ingestion inhibits DTH, CTL lysis, and antigen-specific total IgG induced by traditional systemic vaccines. On the other hand, skin-targeted vaccines were equally immunogenic in alcohol-exposed and non-exposed subjects, suggesting that cutaneous immunization may result in more efficacious vaccination in alcohol-ingesting subjects.

## 1. Introduction

Chronic alcohol abuse increases viral [1,2,3,4,5] and bacterial [6,7,8] infection rates, impairs host immune responses, and can augment co-incident disease progression leading to greater morbidity and mortality. Further, the effectiveness of preventive interventions such as vaccines against hepatitis B [9,10], pneumococcal disease [11], and tuberculosis [12] is reduced in the setting of excessive alcohol use, contributing to observed increases in the frequency and severity of infections.

In attempts to improve the efficacy of traditional intramuscular (IM) vaccinations in alcoholics, high dose and accelerated IM vaccination schedules have been attempted and show some success in improving seroconversion rates in alcohol-consuming non-responders [13,14]. As a potential alternative, intradermal (ID) vaccination has been shown to be dose-sparing and can enhance responses in people who have failed traditional IM vaccinations [15,16]. ID immunization targets the skin immune system, including skin dendritic cells (DCs) and can induce both humoral and cellular immunity, including viral-clearing cytotoxic T lymphocyte (CTL) responses [17]. Since the magnitude of alcohol-induced immune suppression is both dose-related [18,19,20] and tissue-specific [21], skin, an organ with lower alcohol exposure [22], is only moderately affected by alcohol consumption compared to other immune tissues [23,24]. Thus, skin-targeted immunization could result in more efficacious vaccines in alcoholic patients by taking advantage of low skin alcohol levels [22] and an extensive skin DC network.

Modeling the effects of human chronic alcohol consumption remains problematic. Multiple animal models of alcohol exposure are in use, with considerable design variability, including the use of different animal models (including mouse strain), the form and route of EtOH administered, and the amount and duration of feeding; all of which lead to inconsistent results [25]. To evaluate the relative immunogenicity of skin immunization in the context of alcohol ingestion, we chose two commonly employed models differing significantly in the amount of liver damage induced. We reasoned that this approach would address the issue of skin immunogenicity in the setting of alcohol consumption, and the potential impact of concomitant liver dysfunction. Specifically we studied the immunologic impact of chronic alcohol exposure through the relatively “liver sparing” Meadows-Cook (MC) and “liver impairing” Lieber-DeCarli (LD) diets.

It has been established that C57/BL6 mice consume similar quantities of EtOH with both feeding regimes [25]. The MC model incorporates 20% EtOH into drinking water and is combined with standard chow. It is essentially an extended low-stress feeding regime that is associated with very mild to unobservable steatosis and does not increase liver weight or alter corticosteroid levels [26,27]. On the other hand, LD incorporates 4%–6% EtOH with a nutritionally balanced liquid diet that includes 35% dietary fat. These animals are pair-fed with control mice receiving an isocaloric liquid diet with dextrin maltose replacing EtOH. The LD diet causes moderate liver damage including increased liver weight and steatosis [28]. In this model, EtOH consumption generates free radicals and reactive oxygen species (ROS) in the liver, as a direct result of alcohol metabolism and indirectly as a result of gut barrier function impairment [29]. Alcohol consumption inhibits the tight junction proteins occludin and zona occluden, which are critical to the maintenance of intestinal barrier function, which leads to greater permeation of bacterial endotoxin into the circulation [30,31]. Endotoxin can activate Kupffer cells through TLR4 receptor binding [32], which results in secretion of the inflammatory cytokine TNF-α, and increased oxidative stress that is reflected in increased ROS and lipid peroxidation, and decreased hepatocellular antioxidant activity, all of which contribute to steatosis and immune system impairment [33,34].

By utilizing and comparing these two models we sought to determine whether regional immunization targeted to the skin could obviate alcohol induced immune inhibition resulting from direct effects of alcohol on immune function, and/or indirect immuno-modulation by alcohol-damaged liver. To this end, we directly compared immune responsiveness by assessing delayed type hypersensitivity (DTH), antigen-specific cytotoxic T lymphocyte (CTL) activity, and antigen-specific antibody production. Our results suggest that independent of alcohol feeding method, skin immunization obviates alcohol-induced immune suppression observed with systemic immunization, suggesting a novel strategy to improve vaccine effectiveness in alcoholic patients.

## 2. Results

### 2.1. EtOH Levels Are Significantly Less in Skin than in Blood

We previously demonstrated that EtOH administration to rats through either acute (gavage) [35] and/or chronic (LD) feeding regimes [22] leads to significantly greater EtOH levels in blood than in skin. To extend these results to mice, we measured and compared blood and skin EtOH concentrations in groups of mice fed either LD or MC diets. For both feeding regimes, skin EtOH levels are significantly lower than blood levels, supporting the hypothesis that low skin EtOH levels could correlate with relative sparing skin immune function (Table 1). C57/BL6 mice consume similar quantities of EtOH with both feeding regimes [25], thus lower serum levels observed in MC-fed mice may reflect more rapid clearance or earlier cessation of feeding compared to LD (Table 1).

**Table 1:** Alcohol levels vary regionally.

Diet	LD EtOH	MC EtOH	LD Control	MC Control
**Serum EtOH (%)**	0.1380 ± 0.0251 ^a,b^	0.0244 ± 0.0099 ^a,c^	0.0007 ± 0.0006 ^b^	0.0005 ± 0.0003 ^c^
**Skin EtOH (%)**	0.0070 ± 0.0017 ^d^	0.0036 ± 0.0014 ^e^	0.0006 ± 0.0002 ^d^	0.0005 ± 0.0003 ^e^

^a^ *p* < 0.05, ^b^ *p* < 0.0001, ^c^ *p* < 0.05, ^d^ *p* < 0.0001, ^e^ *p* < 0.05.

### 2.2. Alcohol Feeding Protocols Differentially Induce Steatohepatitis and Oxidative Stress

**Figure 1:**
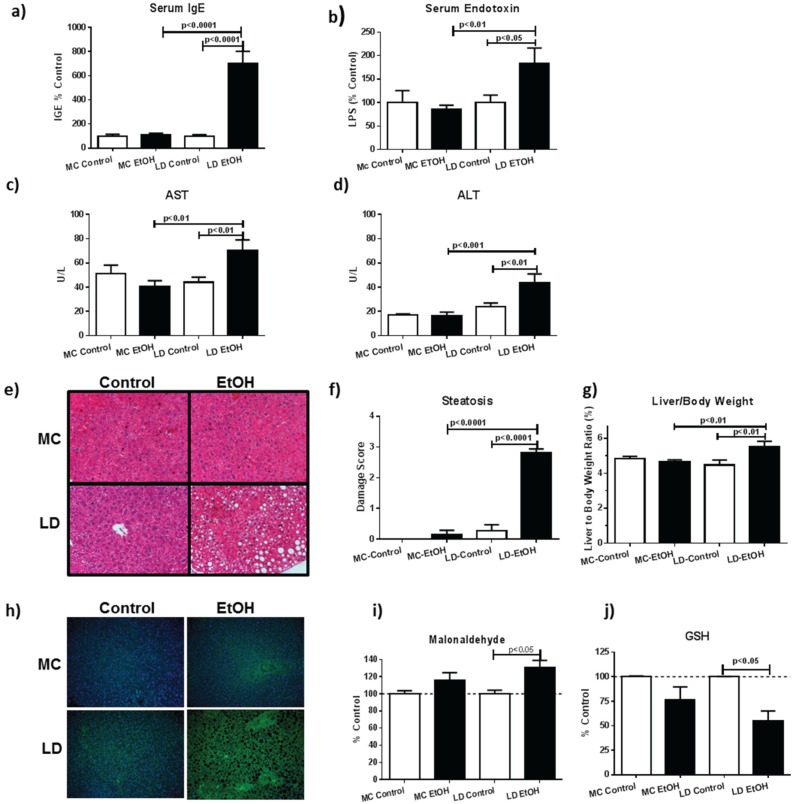
LD EtOH feeding causes greater steatohepatitis and oxidative damage than MC EtOH feeding. Mice were fed alcohol using MC or LD diets. (**a**) Non-antigen specific IgE is increased only after LD EtOH ingestion; (**b**) serum endotoxin levels are elevated only after LD EtOH exposure (*n* = 7–10); (**c**) serum AST and (**d**) ALT are elevated after LD but not MC EtOH exposure (*n* = 6). Livers were weighed and histological sections examined for visual changes due to the feeding models. Representative liver sections stained with H & E (**e**) demonstrate quantitatively more steatosis with LD than MC feeding (**f**) (*n* = 7–11); (**g**) liver weights as % of total body weight are increased after LD EtOH feeding (*n* = 7–13) and lipid peroxidation (4-hydroxynoneal staining) (**h**) is elevated as evidenced by immunofluorescence; (**i**) TBAR assay confirms elevated malonaldehyde in liver homogenates (*n* = 3–8); and (**j**) the antioxidant GSH is significantly depleted after LD diet (*n* = 3–8).

In initial studies we evaluated steatohepatitis and oxidative stress in animals fed LD *vs.* MC diets. We found that non-specific IgE levels indicative of liver damage [36] were elevated from LD but not MC feeding (Figure 1a). Further, serum LPS levels were elevated in LD not MC, indicative of intestinal barrier damage (Figure 1b). In mice fed LD but not MC diets, we found elevated liver enzymes (AST, ALT) (Figure 1c,d), increased liver to body weight ratio (%) (Figure 1g), and histologically-confirmed steatohepatitis (Figure 1e,f)—all direct indicators of liver damage. EtOH metabolism in the liver, including generation of the alcohol metabolite acetaldehyde, generates reactive oxygen species (ROS) leading to oxidative stress [37]. Hydroxyl radicals cause lipid peroxidation, which correlates with levels of reactive malondialdehyde (MDA) and 4-hydroxynonenal (4HNE) [38]. In mice fed LD but not MC, immunohistochemistry specific for (4HNE) demonstrated increased liver lipid peroxidation (Figure 1h), and was supported by direct MDA assay confirming significantly increased lipid peroxidation in the liver homogenate (Figure 1i). The antioxidant imbalance resulting from EtOH metabolism is counteracted by multiple natural antioxidants, including glutathione (GSH), the major non protein thiol present in cells [34]. Consistent with the generation of high levels of ROS, livers from LD fed mice contained less GSH than pair-fed controls (Figure 1j). In all, these results are consistent with previously reported data and support increased liver damage and oxidative stress associated with LD, but not MC EtOH feeding protocols.

### 2.3. Increases in Myeloid Derived Suppressor Cell (MDSC) Populations Correlate with Alcohol Induced Oxidative Stress

**Figure 2:**
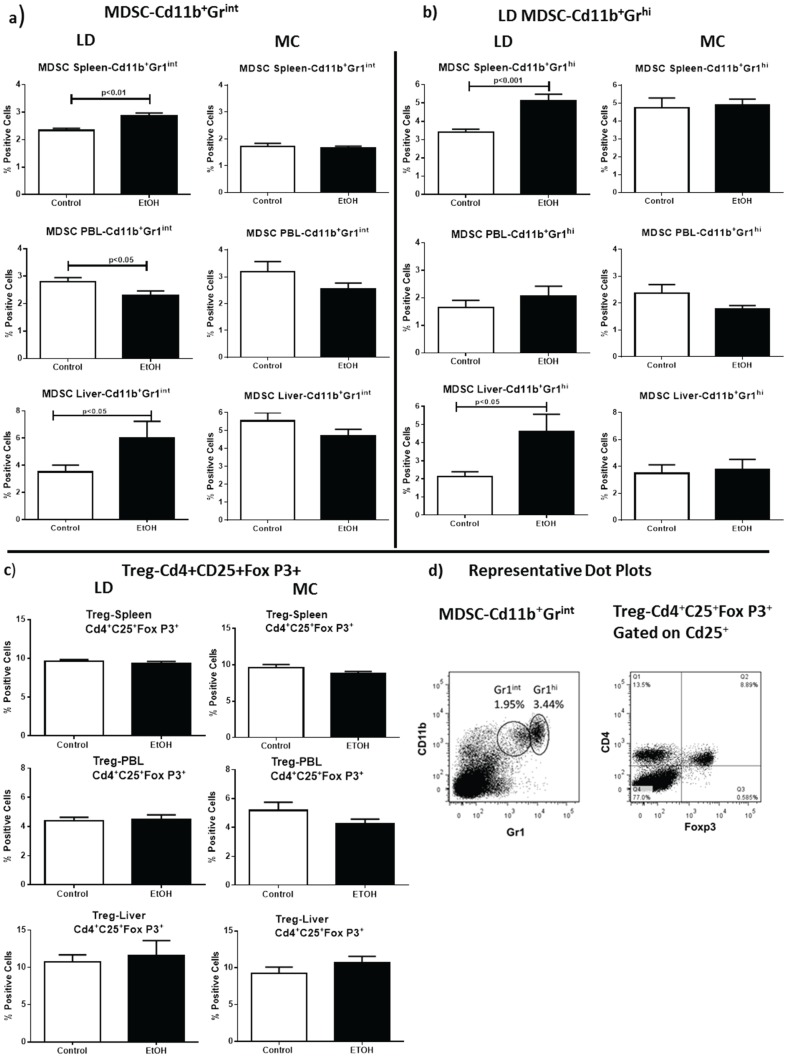
LD Alcohol Feeding Increases MDSC Populations in the Liver and Spleen. (**a**) LD EtOH feeding but not MC feeding induces Cd11b^+^Gr1^int^ and (**b**) Cd11b^+^Gr1^hi^ MDSC populations in the spleen and liver; (**c**) Cd4^+^Cd25^+^Foxp3^+^ Treg populations are unchanged by either EtOH feeding protocol; and (**d**) representative dot plots.

To begin to determine whether increases in oxidative damage observed with LD feeding impacted resident immune cell populations, we quantitated the presence of MDSC and Treg populations in liver, spleen, and peripheral blood leukocytes (PBL). MDSCs suppress effector T cells and regulatory T cell (Treg) populations and have been shown to be up-regulated by multiple factors, including activation of ROS, TLR receptors, STATs, NF-κβ, and iNOS, all of which can be induced by alcohol consumption [37,39,40]. MDSC include at least two phenotypically and functionally distinguishable sub-populations, including CD11b^+^Gr1^int^ and CD11b^+^Gr1^hi^ MDSC populations. These subtypes have been functionally characterized in splenocytes, peripheral blood lymphocytes [41], and liver [42]. Flow cytometry of single cell suspensions demonstrates that both the strongly inhibitory CD11b^+^Gr1^int+^ and mildly inhibitory CD11b^+^Gr1^hi+^ MDSC populations are increased after LD EtOH feeding in liver and spleen (Figure 2a,b). This is in the context of unchanged (CD11b^+^Gr1^hi+^) or modestly decreased (CD11b^+^Gr1^int+^) populations in PBL (Figure 2a,b). Not surprisingly, given the lack of ROS generated by MC, we did not detect significant changes in either MDSC population after MC EtOH feeding. Nor did we detect differences in CD4^+^CD25^+^Foxp3^+^ Treg populations after either EtOH feeding protocol (Figure 2c). The elevated MDSCs measured after LD, but not MC, correlate with increased reactive oxygen species and liver damage observed in our biochemical and histological analysis.

### 2.4. Skin Immunization Obviates Alcohol Associated DTH Inhibition

**Figure 3:**
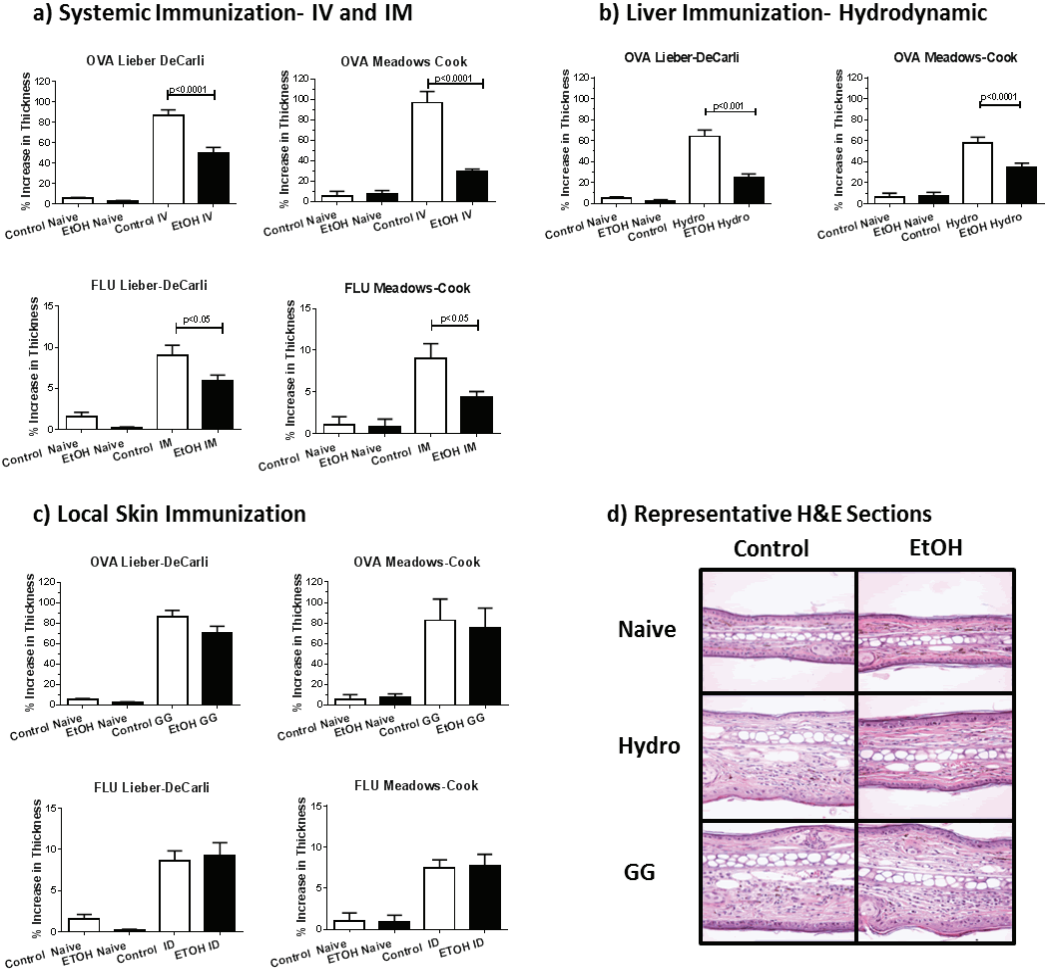
Skin Immunizations Obviate EtOH Induced DTH Inhibition. DTH responses are reported for groups of mice fed by either LD or MC ETOH feeding protocols as indicated after (**a**) systemic; (**b**) liver or (**c**) skin immunization with either OVA pDNA or FLU protein vaccine via intravenous, intramuscular, hydrodynamic, biolistic, or intradermal routes of administration as indicated. DTH responses were elicited and measured as ear or footpad swelling as defined in the methods section. Data are presented as % increase in thickness (ear for OVA, footpad for FLU). LD and MC have equivalent DTH responses; (**d**) representative H and E stained ear sections after OVA immunization and elicitation.

We evaluated the delayed-type hypersensitivity (DTH) reaction as a measure of immune responsiveness to determine the impact of the route of antigen delivery on immune induction in alcohol-consuming mice. To accomplish this, groups of mice were immunized either with plasmid DNA encoding the model antigen Ovalbumin (OVA), or with the protein-based, clinically-relevant, and commercially-available Flu vaccine. Immunization routes included systemic immunization by the intramuscular (IM) or intravenous (IV) routes, or cutaneously by biolistic immunization (gene gun) or intradermal injection (ID). Further, to evaluate hepatic immune function, some groups of mice were immunized intrahepatically by hydrodynamic (HYDRO) injection, a DNA immunization method that results in antigen gene expression in hepatocytes [43]. Using these techniques, we found that DTH responses were inhibited following systemic (IV and IM) and hepatic immunization in animals consuming EtOH compared to non-EtOH consuming animals, regardless of the EtOH feeding protocol (Figure 3a,d). Further, hepatic immunization was substantially less effective in EtOH fed animals (Figure 3b,d). On the other hand, there was no significant difference in the DTH responses induced by skin immunization in EtOH consuming animals *vs.* non-EtOH consuming controls (Figure 3c,d). This was consistently true regardless of the EtOH feeding protocol, DNA *vs.* protein immunization, or GG *vs.* ID delivery. Thus, regardless of feeding protocols, reduced immunogenicity of traditional systemic protein and DNA vaccines observed in EtOH-consuming animals was not evident for skin-targeted immunizations.

### 2.5. Skin Immunization Overcomes Alcohol Induced Inhibition of CTL Induction

**Figure 4:**
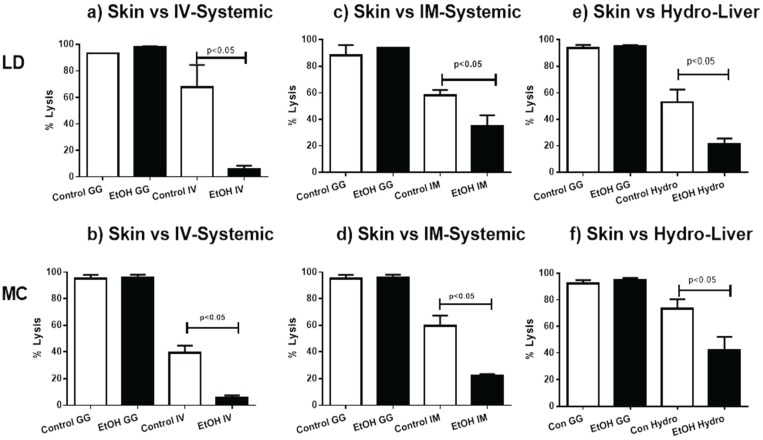
Skin Immunization Obviates EtOH Induced Inhibition of CTL Induction. Groups of animals fed ETOH by LD (**a**,**c**,**e**) or MC (**b**,**d**,**f**) and their matched non-EtOH-fed controls were immunized by IV (**a**,**b**); IM (**c**,**d**); or HYDRO (**e**,**f**) routes as previously described and in each case compared to biolistically (GG) immunized paired EtOH-exposed and control mice biolistic. Immunization protocols were as previously described and CTL induction was determined by *in vivo* lytic activity and presented as % lysis as defined in the methods section. Representative experiments are shown (*n* = 2–9).

As alcoholics are more susceptible to infection and cancer, we sought to determine the effect of alcohol consumption on the induction of CTL responses by cutaneous or systemic vaccines, as measured by *in vivo* antigen-specific lytic activity. The induction of lytic responses was inhibited by EtOH consumption for vaccines delivered by IV, IM or hydrodynamic routes regardless of the method of EtOH feeding (Figure 4). In contrast, EtOH-consuming animals immunized cutaneously demonstrated strong antigen specific *in vivo* lytic activity that was equivalent to that observed in control EtOH naïve animals (Figure 4). This was consistent across feeding protocols. To ensure that potential EtOH effects were not masked by the high magnitude of antigen-specific lysis induced, we repeated skin immunizations without boosting, which resulted in more moderate degrees of lysis without differences between EtOH-consuming and naïve animals [44]. Thus, regardless of EtOH feeding protocols, EtOH associated inhibition of antigen-specific lytic cell induction was evident following systemic immunization, but avoided by skin-targeted antigen delivery.

### 2.6. Skin Immunization Obviates Alcohol Induced Inhibition of Antigen-Specific IgG Induction

**Figure 5:**
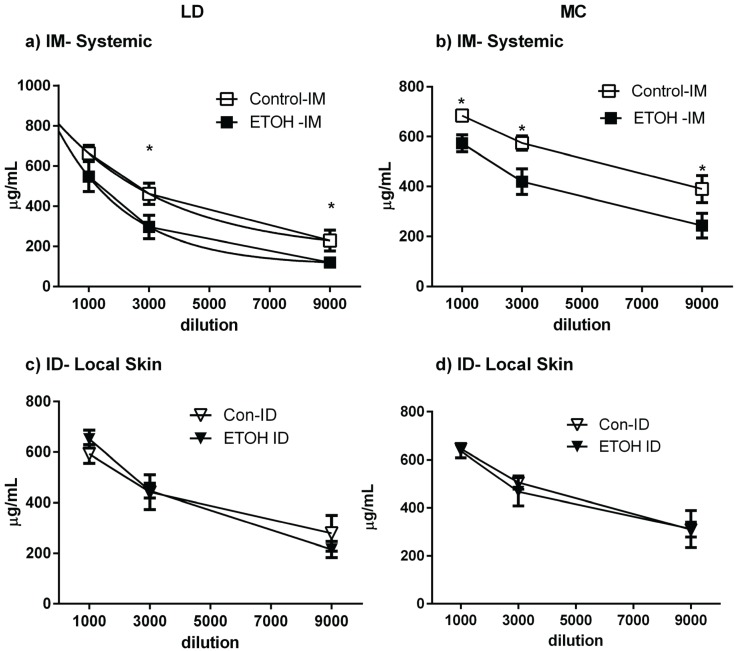
Skin Immunization Obviates EtOH Induced Inhibition of Vaccine Induced IgG. Mice fed ETOH by LD (**a**,**c**) or MC (**b**,**d**) protocols and EtOH-naïve controls were immunized with influenza vaccine by IM (**a**,**b**) or ID (**c**,**d**) routes and boosted on day 21 (*n* = 3–8). Total antigen-specific IgG, as determined by Elisa, is shown, with * *p* < 0.05.

EtOH ingestion is known to impair vaccine efficacy in humans, as measured by antibody responses including antigen-specific IgG [12]. Using influenza vaccine, we evaluated antigen specific IgG responses in both EtOH-consuming and EtOH-naïve groups of animals as a function of immunization route. EtOH consumption, whether by LD or MC protocols, significantly inhibited antigen-specific IgG responses elicited by IM immunization (Figure 5a,b). In contrast, there was not a significant difference between EtOH-consuming mice and naïve controls when the flu vaccine was delivered ID (Figure 5c,d). This was observed regardless of EtOH feeding protocol, suggesting that skin-targeted immunization can elicit effective IgG responses even in the setting of chronic alcohol ingestion.

## 3. Discussion

Considerable evidence from animal models and human studies suggests that chronic alcoholism is associated with clinically-relevant immune dysfunction [1,2,3,4,5,6,7,8]. Mechanisms of alcohol-induced immune suppression are complex, and depend on both direct effects of alcohol exposure on immune cells, as well as downstream effects of alcohol metabolism including, for example, the generation of acetaldehyde and ROS. Adding to this complexity are the myriad immunosuppressive effects of steatohepatitis and liver failure that are frequent sequelae of chronic alcoholism. Alcohol-related immune suppression is both tissue specific [21] and dose dependent [19,20]. We hypothesized that a significant component of immune inhibition resulting from alcohol consumption could be related to direct exposure of immune cells to alcohol and its metabolites. This is consistent with *in vitro* models demonstrating alcohol’s inhibitory effects on dendritic cell function [45,46,47]. Given previous studies demonstrating relatively low levels of alcohol in skin compared to other organs [22,35], we reasoned that the skin immune system may be relatively spared from the immune-inhibitory effects of alcohol.

To test this directly, we compared the immunogenicity of traditional systemic vaccination strategies to skin targeted immunizations. Antigen was targeted to the systemic circulation (IV), muscle (IM), the liver (hydrodynamic delivery) or the skin (gene gun, ID), and immunogenicity was determined by evaluation of DTH, CTL, and humoral immune responses. Further, to more specifically address immune-suppressive effects from direct alcohol exposure *vs.* alcohol induced hepatic dysfunction, we studied these immunization strategies in models of chronic alcohol ingestion with, and without, hepatic dysfunction.

The potential effectiveness of skin vaccination in heavy alcohol users is supported by our results demonstrating that both DTH and CTL responses are inhibited by alcohol ingestion after systemic and hepatic immunization, but not after skin immunization. The skin immune system induces both the cellular and humoral arms of immunity, thereby allowing for the effective development of viral clearing CTL [17]. Our observation that alcohol ingestion inhibits DTH, CTL, and antibody responses independent of feeding model, is consistent with previous studies in both LD [48] and MC mice [49]. The observation that skin immunization overcomes alcohol inhibition in both models is encouraging for clinical extrapolation. To that end, inhibition of DTH responses by alcohol is well established in human subjects [50]. Kinetic studies using CTL and DTH assays reveal that immune suppression is strongest when alcohol ingestion occurs before or during sensitization [51,52,53], and may be reversible after EtOH cessation [54]. Further, in humans hemagglutinin inhibition of antibody following IM influenza vaccination is inhibited in patients with advanced liver cirrhosis [55], and alcohol dependency significantly reduces hepatitis B vaccine efficacy after IM delivery [56]. Finally, our results are consistent with reports that ID influenza vaccination is more effective than IM immunization in older adults [57] and immunosuppressed patients [58,59,60]. Thus, skin immunization has potential to effectively obviate poor seroconversion rates observed following IM vaccination in heavy alcohol users.

Mechanistically, it is well-established that alcohol interferes with the ability of DCs to activate T cells after antigen stimulation [61]. Alcohol interferes with DC maturation, resulting in decreased expression of the costimulatory surface molecules (CD40, CD80, CD86) important for T-cell activation [62,63]. It has also been reported that MHC class I and class II expression is decreased [48,62], and that these differences are associated with reduced secretion of inflammatory cytokines such as TNFα, IL-12, IFNγ, IL6 and IL-17A, and enhanced expression of IL-1β, IL-10 and IL-13 by ethanol-exposed DCs [48,53,64].

Alcohol effects on DC function also appear to be regional. Chronic alcohol ingestion decreases DC numbers in the spleen, but increases DCs in thymus [23]. Alcohol also induces hepatic DC migration to draining lymphoid tissue, but does not affect splenic DC migration. Hepatic DCs prime allogeneic cells more vigorously than splenic DCs after alcohol exposure. DC populations in the skin, with relatively low levels of alcohol exposure [22], appear to be only moderately affected by alcohol consumption. For example, four weeks of 20% EtOH in mouse drinking water leads to a 30% reduction in Langerhans Cells (LCs), but no change in dermal dendritic cell (DDC) numbers. In contrast, total splenic DCs are reduced by 50% under the same conditions. Migration of LC and dermal DC from the skin to draining lymph nodes during inflammation is modestly delayed in alcohol-fed compared to control mice. LC are more sensitive to the effects of alcohol compared to dermal DC, as shorter durations of alcohol exposure are required to inhibit migration [23,24,47]. The relative sparing of dermal DC function may contribute to the preservation of skin immune function.

Tregs and MDSCs have inhibitory effects on antigen specific CTL induction and vaccine efficacy [65,66]. Previous studies suggest that MDSCs are increased in PBL after alcohol ingestion compared to normal controls [67]. Our data suggest that under non-pathological conditions, MDSC populations were increased in both the spleen and liver from the LD diet. The MC diet did not substantially alter MDSCs. Treg populations were not affected by either alcohol diet. The differences between MC and LD diets suggest that MDSC induction is likely an effect of alcohol associated liver dysfunction rather than a direct effect of alcohol itself. In any case, the minor changes observed did not appear to effect either alcohol associated immune suppression or the capacity of skin immunization to overcome it.

To address the impact of moderate liver damage on vaccination in mouse models of alcohol consumption, we directly compared the steatohepatitis-generating LD with the less-damaging MC diet. Direct comparisons between the two models are rarely performed. Histological and quantitative data demonstrate steatohepatitis after LD, but only minimal changes in liver morphology after MC. Our data suggests that only LD disrupts small intestine barrier function, as indicated by elevated serum LPS consistent with previous observations in mouse models [68] and humans [31]. All markers tested confirm extensive oxidative stress in LD, but not MC-fed mice, consistent with previously published studies [34,69,70]. The differences in inflammation and oxidative stress enabled us to address the impact of these stressors on immune function. These studies also provide a direct comparison of the liver damaging LD *vs.* liver-sparing MC diet on systemic immune responses in C57/BL6 mice, and suggest that alcohol induced immune suppression did not depend on increased serum LPS, steatohepatitis, or oxidative stress as measured by increased lipid peroxidation and decreased GSH in liver by LD, but not MC. Thus, in these models, alcohol-related immune suppression appears to be directly related to the direct effects of alcohol and its metabolites and not to indirect effects of hepatic dysfunction. It is important to note, however, that these mouse models correspond to moderate alcohol-induced liver damage, but do not model liver cirrhosis which may produce more severe immune dysfunction.

## 4. Experimental Section

### 4.1. Feeding Regimes

Female C57BL/6 mice (6–8 weeks old) were fed a nutritionally-adequate LD liquid diet [28] consisting of 18% protein, 37% fat, and 47% carbohydrate containing 5% (*v*/*v*) EtOH (27% calories), or a control diet in which EtOH is substituted isocalorically with dextrin maltose (Bio-Serve, Frenchtown, NJ, USA). EtOH was introduced gradually by increasing the content from 1% for two days to 3% for two days and then 5% (*v*/*v*) ethanol for the remainder of the experiment. Mice were given 5% EtOH for one, four or eight weeks prior to immunization and remained on 5% EtOH for the entire experiment.

A separate set of female C57/BL6 mice (6–8 weeks old) were provided EtOH in drinking water combined with chow ad libitum (MC). Mice were acclimatized to alcohol by ramping up the concentration starting at 10% for two days, increasing to 15% for five days and 20% for 6–8 weeks at which time they were immunized and remained on 20% EtOH for the duration of the experiment.

### 4.2. Immunization Methods

Ovalbumin pDNA was administered either via intramuscular (IM) or intravenous injection (IV), hydrodynamic injection (targeted to liver) or biolistic delivery (targeted to skin). The high pressure/high volume hydrodynamic immunization (Hydro, enables pDNA delivery directly into hepatocytes by rapid intravenous administration (6–8 s) of a large volume of DNA (12 μg of plasmid OVA DNA in 1.5 mL of PBS) [43]. Skin delivery was achieved with a biolistic gene gun system. Twelve μg of plasmid OVA DNA was coated onto microscopic gold particles (1–3 μm in size), which were then delivered to the abdominal skin using a helium-powered GG (Helios, BioRad Laboratories, Hercules, CA, USA) at a helium pressure of 250 PSI (GG) [71]. For systemic delivery, we used IV or IM immunizations of 12 μg of plasmid OVA DNA in 0.2 mL of PBS.

Fluvirin (207–2008 formulation, Novartis, East Hanover, NJ, USA) (FLU), a purified split virus preparation influenza vaccine, containing A/Wisconsin/67/205, A/Solomon Islands/3/200, and B/Malausia/2506/2004 hemagglutinin antigens (HA), was delivered to mice using IM or ID injections at a dose of 3 μg.

### 4.3. Serum and Tissue Characterization

Upon completion of immunization protocols, shaved skin (20 mg) was homogenized for 75 seconds in 300 μL of PBS and spun for 10 minutes. We assayed the supernatant for skin EtOH with a L3K kit (Sekisui Diagnostics, Framingham, MA, USA). Serum EtOH, AST, ALT, and endotoxin were also determined with commercial kits (L3k, AST-SL, ALT-SL, Sekisui Diagnostics, 50-647U Lonza, Allendale, NJ, USA). Non-antigen-specific IgE levels were measured via ELISA (BD Opt EIA, BD Biosciences, San Jose, CA, USA). Data are presented as % control for each feeding model and statistical significance determined with an ANOVA followed by Fisher Exact Test with significance set at *p* < 0.05 (GraphPad Prism, GraphPad Software, Inc., La Jolla, CA, USA).

H & E-stained paraffin embedded liver sections from non-immunized mice were evaluated for steatosis, by a blinded hepatologist, with 0 = <5%, 1 = 5%–33%, 2 = 33%–66%, 3 = >66% [72]. Statistical significance was determined with an ANOVA followed by Fisher Exact Test with significance set at *p* < 0.05 (GraphPad Prism).

### 4.4. Liver Biochemical Assays

Liver samples were homogenized in 25mM HEPES, 1mM EGTA, 5mM MgCl_2_ on ice and spun at 13k rpm for 15 min at 4 °C [73]. We tested supernatant for GSH (Gsh-Glo, Promega, Madison, WI, USA) and lipid peroxidation (Oxiselect TBARS Assay Kit, Cell BioLabs, San Diego, CA, USA). Protein levels were determined with Coomassie Plus Protein Reagent (Fisher Scientific, Pittsburgh, PA, USA). Data are normalized to mg protein and are presented as % control diet. Statistical significance was determined with an ANOVA followed by Fisher Exact Test with significance set at *p* < 0.05 (GraphPad Prism).

### 4.5. 4HNE Immunohistochemistry

Slides were deparafinized, rehydrated, washed in PBS, and stained with Polyclonal Goat anti 4-HNE primary antibody 1/250 in 1% BSA in PBS 0.1% TritonX-100 (Alpha Diagnostic, San Antonio, TX, USA) overnight at 4 °C, followed by conjugated Donkey anti-Goat IgG cy2 secondary antibody (Jackson Immuno Research, Ann Arbor, MI, USA) and counterstained with DAPI [74].

### 4.6. Myeloid Derived Suppressor Cells (MDSC) and Regulatory T Cells (T_reg_)

Peripheral blood leukocytes (PBL) were purified from blood according to manufacturer instructions using Lympholyte-Mammal Cell Separation Media (Cedarlane, Burlington, ON, Canada). Livers and spleens were homogenized and filtered. Livers were then suspended in a 35% Percoll gradient (Sigma Chemical, St Louis, MO, USA) and centrifugation for 15 min at 450 g at room temperature [75]. RBCs were lysed from spleens and livers prior to staining. Cells were stained using anti-CD4 (560782, clone: Rm4-5), anti-CD25 (551071, clone: PC61), anti-CD11b (557657, clone: 557657), anti-Ly6g (557657, clone: M1/70) (BD Biosciences), and anti-FoxP3 (17-5773-82, clone: FJK-16s, eBiosciences, San Diego, CA, USA) antibodies were diluted with 10% goat serum in PBS and incubated for 45 min on ice. Cells were permeabilized using FoxP3 staining buffer set (eBioscience) following manufacturer instruction, except cells were incubated in the fix/permeabilization media overnight at 4 °C. Anti-FoxP3 antibody was diluted in permeabilization buffer and added to cells for 30 min at room temperature. Staining was determined via flow cytometry. Statistical significance was determined via an unpaired *t*-test with *p* < 0.05 (GraphPad Prism).

### 4.7. Delayed Type Hypersensitivity (DTH)

We compared DTH responses between EtOH and pair-fed mice after immunization with either OVA pDNA or influenza vaccine. OVA mice were immunized on Day 1 and boosted on Days 7 and 14 [76]. On Day 19, an eliciting dose was applied by GG to one ear. Ear thickness was measured 24 h. In FLU DTH experiments, mice were immunized either IM or ID with 3 μg influenza vaccine on Day 1 and were boosted on Day 21. One week later, vaccine was injected into one footpad and PBS into the other. Footpad thickness was measured at 24 h. All data are presented as % increase in thickness, determined using the equation:

(1) $$\left(\frac{\text{Treated Thickness }–\text{ Control Thickness}}{\text{Control Thickness}}\right)\times 100$$

Statistical significance was determined with an ANOVA followed by Fisher Exact Test with significance set at *p* < 0.05 (GraphPad Prism).

### 4.8. In Vivo Cytotoxic T Lymphocyte Killing Assay (CTL)

To evaluate *in vivo* antigen specific lytic activity as a measure of CTL activity, mice were immunized with OVA pDNA via GG, IV, IM, and Hydro. Boosts were given on Days 7 (all), 14 (IV and IM), and 28 (IM) [77]. An *in vivo* CTL assay was performed five days after the last boost [76]. Percent lysis was calculated using the formula [76]:

(2) $$\text{\% lysis }=\;100\text{ x }1-\left(\frac{\;\frac{\text{CFSElow}}{\text{CFSEhigh}}\text{of splenocytes from untreated mice}}{\;\frac{\text{CFSElow}}{\text{CFSEhigh}}\text{of splenocytes from immunized mice }}\right)$$

Statistical significance was determined via a one sample *t*-test and are compared to a theoretical mean of 100 (*p* < 0.05) (GraphPad Prism).

### 4.9. Antibody Measurements

ELISA plates were coated with influenza vaccine (2 μg/mL). Plates were blocked with 1% BSA in PBS for 1 h, followed by biotinylated goat anti-mouse Fc receptor specific total IgG (Jackson Immuno, West Grove, PA, USA) for 2 h and Avidin-HRP (BD Pharmingen, San Jose, CA, USA) and then developed with TMB. The reaction was stopped immediately by adding 0.3 M Sulfuric Acid. Data were converted via a standard curve generated with IgG (Sigma, I5381). Plates were read at 450 nm. Statistical significance was determined via an unpaired *t-*test with significance set at *p* < 0.05 (GraphPad Prism).

## 5. Conclusions

The long-term goals of these efforts are to better understand the effects of alcohol consumption on skin immunity, and to improve vaccine efficacy in alcohol consuming patients. We demonstrate that skin immunization obviates alcohol-induced immune inhibition in both liver sparing and liver-impairing mouse models of chronic alcohol consumption. Improvements in vaccine responses in heavy alcohol users could substantially increase vaccine efficacy in this large, well-defined population, with significant downstream public health benefits in terms of morbidity, mortality, and healthcare costs. This work supports the concept of ID vaccination as a standard of care for alcoholics.
